# Different mitogenomic codon usage patterns between damselflies and dragonflies and nine complete mitogenomes for odonates

**DOI:** 10.1038/s41598-018-35760-2

**Published:** 2019-01-24

**Authors:** De-Long Guan, Zeng-Qiang Qian, Li-Bin Ma, Yi Bai, Sheng-Quan Xu

**Affiliations:** 1 College of life science, Shaanxi Normal University, Xi’an, 710119 P. R. China; 2 School of Life Science, Taizhou University, Taizhou, 317000 P.R. China

## Abstract

Damselflies and dragonflies, of the order Odonata, have distinct body plans and predatory abilities. Knowledge of their various evolutionary histories will allow for an understanding of the genetic and phenotypic evolution of insects. Mitogenomes are suitable materials to elucidate this, but the mitogenome of only a few odonates have been annotated. Herein, we report the complete mitogenome of nine odonates, including seven dragonflies and two damselflies, and a comprehensive analysis of the codon usage in 31 Odonata mitogenomes with the aim to estimate their evolutionary characteristics. Overall, a weak codon bias exists among odonate mitogenomes, although this favours AT-ending codons. Damselflies have a weaker codon usage bias than dragonflies, and 37 codons have significantly different usages. Both directional mutation and purifying selection shape damselfly and dragonfly mitogenomes. Although inevitable, directional mutation bias plays a minor role, whereas purifying selection pressure is the dominant evolutionary force. A higher selection pressure is observed in dragonflies than in damselflies, but it mainly acts on codon usage patterns rather than amino acid translation. Our findings suggest that dragonflies might have more efficient mitochondrial gene expression levels than damselflies, producing more proteins that support their locomotion and predatory abilities.

## Introduction

The order Odonata comprises three suborders: dragonflies (Anisoptera), damselflies (Zygoptera), and the unique endangered ancient dragonflies, the Anisozygoptera^1–3^. These insects are hemimetabolous, and are characterised by highly developed compound eyes and two pairs of long wings that move independently^1,4,5^. Most odonates are carnivorous, except for the adults of a few species that feed on fruits (e.g., *Lestes temporalis*)^3,4^. The larvae are aquatic predators and adults feed mostly on small flying insects^1,3,6^. Odonates are highly vulnerable to wetland degradation as their larvae depend on freshwater habitats for food, and therefore, the presence of odonates can be used as an indicator of water quality^3,4,7,8^.

Although odonates share similar slender body plans, dragonflies differ from damselflies in several characters^9,10^. Dragonflies are generally larger and hold their wings horizontally when at rest, whereas damselflies are smaller, and most species fold their wings over the abdomen when stationary^9,10^. The eyes of dragonflies are never more distant than the distance between the antennae. However, the most striking difference between dragonflies and damselflies is their locomotion ability^5,10–12^. Dragonflies are powerful fliers, which can migrate across great distances, with great speed and agility. Some dragonflies, such as *Anax* and *Libellula* species, can fly forward at over 10 m per second. Even with a high power/weight ratio, most damselflies fly only short distances and for short periods^12–14^. As flight is their primary mode of locomotion, the difference should originate from different evolutionary constraints during their long evolutionary history. Flying requires an adequate source of energy delivered by mitochondria and therefore mitochondrial genes of damselflies and dragonflies might have been the target of different evolutionary constraints^15–19^.

Mitochondria are semi-autonomous organelles, possessing a specific genome (mitogenome); the mitochondrial genes have been extensively used as molecular markers in phylogenetic, conservation, and evolutionary studies. Originally, the mitogenome was considered to be neutral^20^, but recent studies have provided new evidence that mitogenomes, like nuclear genomes, also underwent adaptive evolution. Some factors such as thermal environment (climate or body-heat), predatory behavior (diet), and locomotion have been considered to influence the evolution of mitogenomes^15,21–31^. Obviously, flight is associated with the molecular evolution of insects, and energy consumption in flying insects, such as beetles and fruit flies, has led to the adaptive evolution of mitochondrial genes^15,16,18,19,29,32–34^.

Considering the life-style and ecological niche of odonates, their flight mechanism is considered to be essential for their survival and evolved most likely under strong adaptive pressure. A better understanding is crucial for the interpretation of the evolutionary path and history of odonates and other winged insects^2,14,18,33,35^. However, the complete mitochondrial sequences required to infer the evolutionary events affecting Odonata lineages are scarce; only 22 complete or nearly complete mitogenome sequences are known and every additional sequence would be a step forward in Odonata research.

The importance of evolutionary forces, including mutation bias, selective pressures, and genetic drift, has been revealed by features such as nucleotide content bias, transcription and translational efficiency of genes, and the number of accumulated adaptive or fixed deleterious mutations^36–40^. Although determining and comparing the extent of these features require multiple measurements, they could be easily combined in codon-usage pattern examinations. Protein coding genes occupy over 70% of the total mitogenome length, and codon translations are suitable to determine whether changes in the mitogenome are synonymous^20,41^. Moreover, in insect mitogenomes, all amino acids are encoded by at least two types of codons, which are unevenly used, and different genes and species may exhibit different patterns as an adaption to environmental pressures^21,39,42^. Thus, characterising the presence and pattern of codons will help understand the process of long-term evolutionary forces and the balance between mutational bias and selection in the formation of mitogenome of dragonflies and damselflies.

In the present study, we examined the mitogenome of nine odonates by next-generation sequencing. Furthermore, we reconstructed the complete mitogenome of seven Anisoptera dragonflies (*Tramea virginia*, *Orthetrum testaceum*, *Orthetrum sabina*, *Orthetrum melania*, *Deielia phaon*, *Acisoma panorpoides*, and *Trigomphus carus*) and two Zygoptera damselflies (*Atrocalopteryx atratum* and *Platycnemis foliacea*). The mitogenome features of these two species have been described. The codon usage bias in protein coding genes within the mitogenome of dragonflies and damselflies was examined to reveal the evolutionary forces that contributed the most in shaping the mitogenomes and their codon usage patterns in dragonflies and damselflies.

## Results

### Nine newly characterised complete mitogenomes

**Figure 1:**
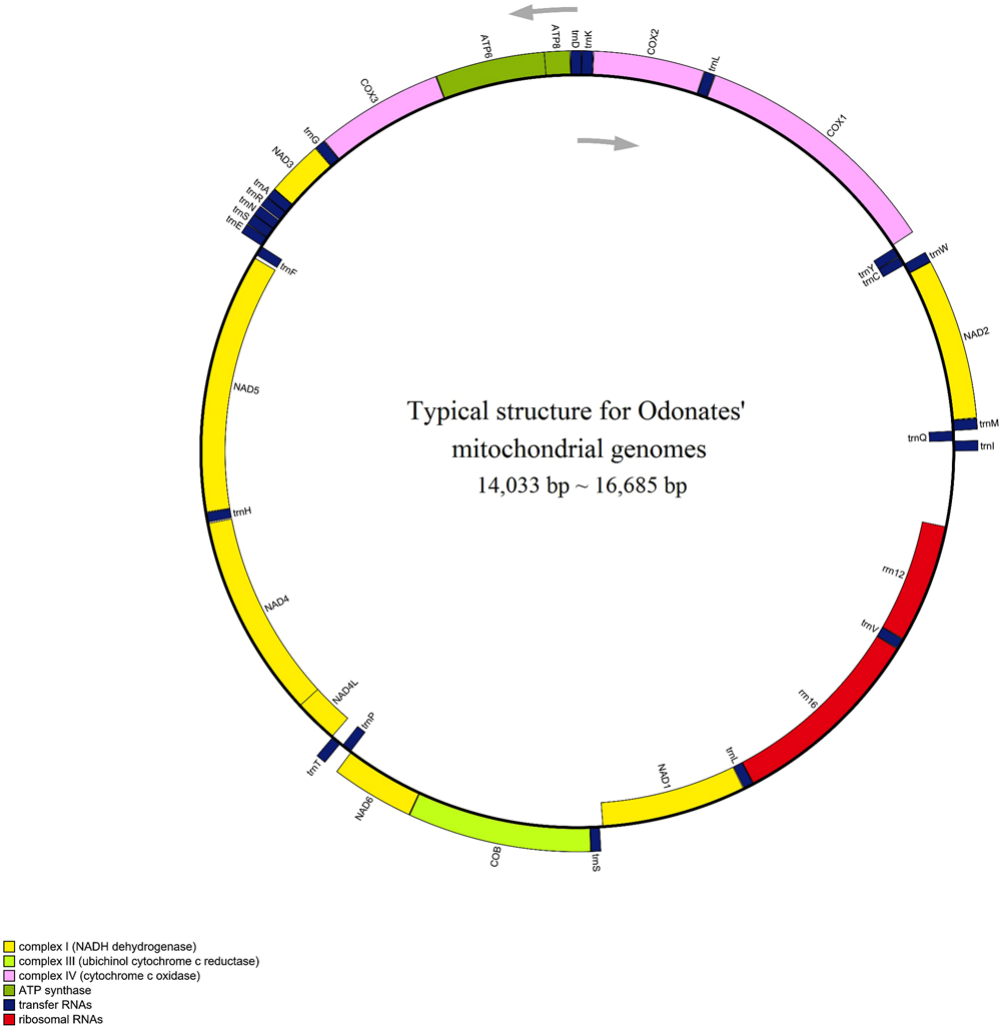
Typical physical map of the mitochondrial genome of odonates. Protein coding, transfer RNA (tRNA), and ribosomal RNA (rRNA) genes are indicated in green, pink, and red, respectively.

The attributes of the mitogenome of dragonflies and damselflies are very similar; they have similar lengths, the same gene content and arrangements, and the same trend of AT-bias. Currently available Odonata mitogenomes are 14,033 bp (*Orthetrum melania*) to 16,685 bp (*Vestalis melania*) long^43–46^. The mitogenomes reported herein are within this range: *T*. *carus* is the shortest with 15,135 bp and *A*. *panorpodies* is the longest with 15,742 bp. Each mitogenome consists of 37 genes, including 13 protein-coding genes (PCGs), 22 transfer RNAs (tRNAs), two ribosomal RNA (rRNA) genes, and one control region (CR) (Fig. 1). These genes are arranged as in other odonates, and most of them (9 PCGs and 14 tRNAs) are on the majority strand^43–46^. The nucleotide composition of these mitogenomes is strongly biased toward A and T, similar to that observed in other odonates^43,44,46^; the combined AT content was above 70%. Mutations in odonates are directional (preferably toward the AT duplex bond). Similar to that in most other insects, the mitogenome of odonates reported herein have a long AT-rich non-coding region. The CR, which is the original replication region for both strands of the mitogenome, is considered hidden within the AT non-coding region. These new mitogenomes will be helpful for future evolutionary studies, and the PCGs of *A*. *atratum* and *P*. *foliacea* were used to determine the codon usage patterns in Odonata mitogenomes.

### Codon usage patterns in the mitogenomes of dragonflies and damselflies

**Figure 2:**
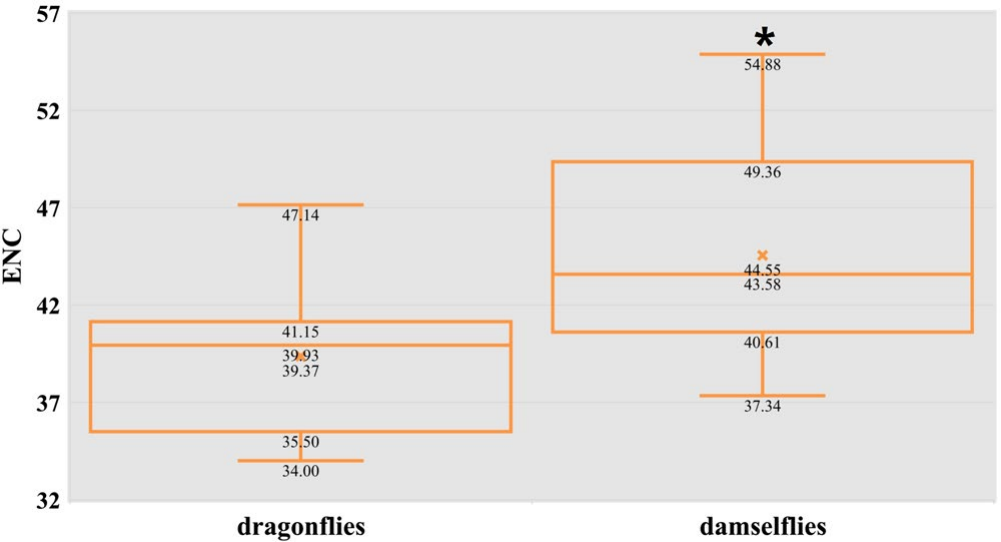
Box-plots of the effective number of codons (ENC) in dragonflies (left) and damselflies (right). The asterisk (*) indicates that there was a significant difference between the two groups (*t*-test, *P* < 0.05).

The extent of codon usage bias in the mitogenome of dragonflies and damselflies was determined and compared based on the effective number of codons (ENC) and relative synonymous codon usage (RSCU) values of 31 Odonata species. The values obtained for ENC (Fig. 2) ranged from 34.00 to 54.88, with an average of 41.37. Using the conventional ENC < 35 to delineate strong codon usage, this average value indicated a generally weak codon usage bias in Odonata mitogenomes. The newly surveyed species presented their own patterns of synonymous codon preferences with the ENC values above 35. The ENC values of damselflies were significantly higher (*t*-test, *P* < 0.05) than those of dragonflies, ranging from 37.34 to 54.88 and from 34.00 to 47.14, respectively. Thus, damselflies tend to use more types of codons to produce proteins, suggesting that their mitochondrial genes might undergo weaker selection constraints with respect to replication speed and transcription efficiency and accuracy. As gene expression level account for these mechanisms, the basal energy metabolism in damselflies might be less efficient than that in dragonflies.

**Figure 3:**
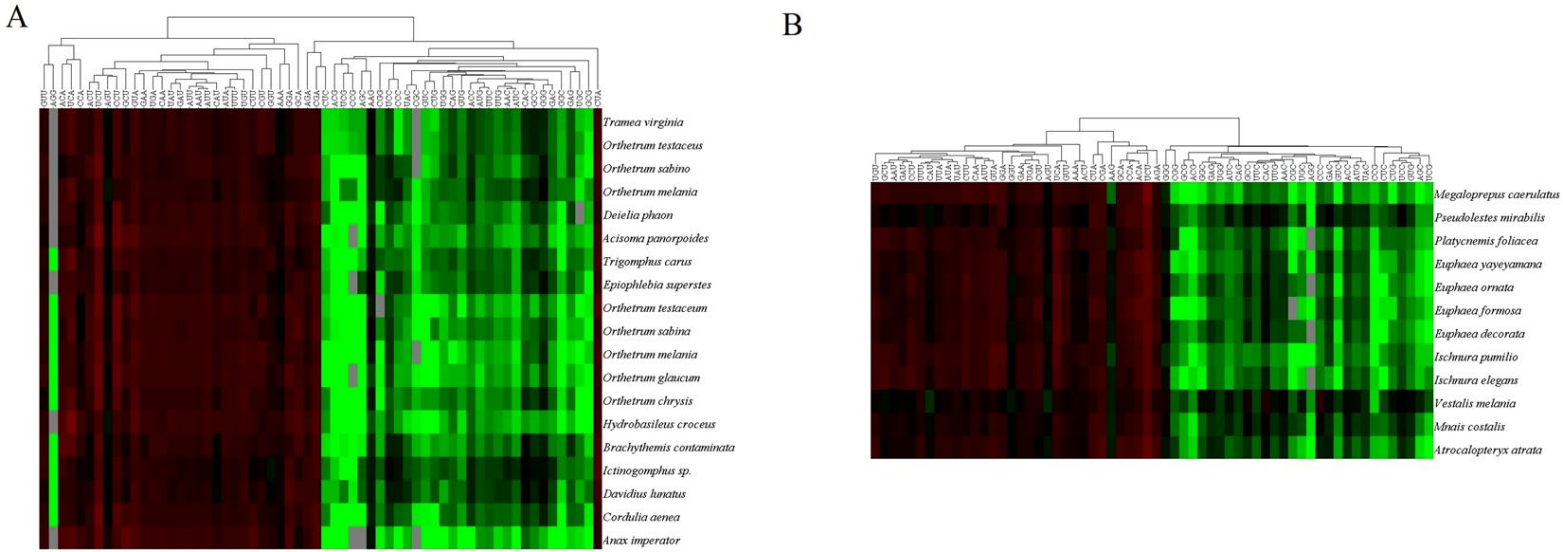
Hierarchical cluster analysis and heat maps of the relative synonymous codon usage (RSCU) values of each codon in the mitogenome of dragonflies (**A**) and damselflies (**B**). Each square in the heat map represents the log ratio of the RSCU value of each codon (in columns) within the mitogenome of each Odonata species (in rows). Colours indicate the magnitude of RSCU values: black, RSCU = 1 (no bias in codon usage); green RSCU < 1; red, RSCU > 1; grey, no data.

The RSCU values of each codon within the 31 Odonata mitogenomes analysed are provided in Supplementary Tables S1. Almost all the 62 codons found were present in the 31 species, except for AGG, CCG, and CGC that were absent in some. In particular, *O*. *glaucum*, *A*. *imperator*, and *E*. *superstes* do not use CCG; *O*. *melania*, *A*. *imperator*, and *E*. *formosa* do not use CGC; six species, including *H*. *croceus*, *A*. *imperator*, *E*. *superstes*, *E*. *decorate*, *E*. *ornate*, *I*. *elegans*, and *P*. *foliacea*, do not use AGG. The codon UUA presented the highest RSCU values, whereas AGG was the most rarely used codon; 29 codons were high-frequency (RSCU > 1) codons and the remaining were low-frequency codons. To determine and compare the RSCU trends in dragonflies and damselflies, the values were clustered separately and two heat maps were obtained (Fig. 3A,B), with two main groups: preferential codons (represented in red) are mainly distributed on the left branches of the cluster diagram, whereas the less-preferred codons (represented in green) are mostly distributed on the right branches. Almost all preferentially used codons are AT-ending, which is consistent with that observed in several AT-rich genes and organisms, and is a simple reflection of their overall nucleotide components. Biased nucleotide compositions, which are strongly influenced by directional mutation bias, will impose restrictions on the combination of triplets (codons). Specifically, substitutions in the third position of codons alter the RSCU as they translate into synonymous amino acids, whereas substitutions in the other two positions change codon translations, and do not interfere with the RSCU calculation.

The trends of RSCU in dragonflies and damselflies exhibited a slight inconformity. The AT- and GC-ending codons were more clearly separated in damselflies, the RSCU of the four AT-ending codons (GGA, AGA, AAA, and GUU) presented similar usage pattern, whereas the codons CCG and AGG are rarely present in dragonflies. As clusters indicate the similarity between two sets of values, these results indicate that for some codons in the mitogenome of dragonflies, a statistical correlation may emerge from the dominant similarity instead of disparity between values. The usage of different types of highly preferred codons observed in dragonflies suggests that they might have experienced more specific evolutionary forces than that in damselflies and that the codons were selected based on usage preference in addition to nucleotide components, indicating selective constraints.

**Figure 4:**
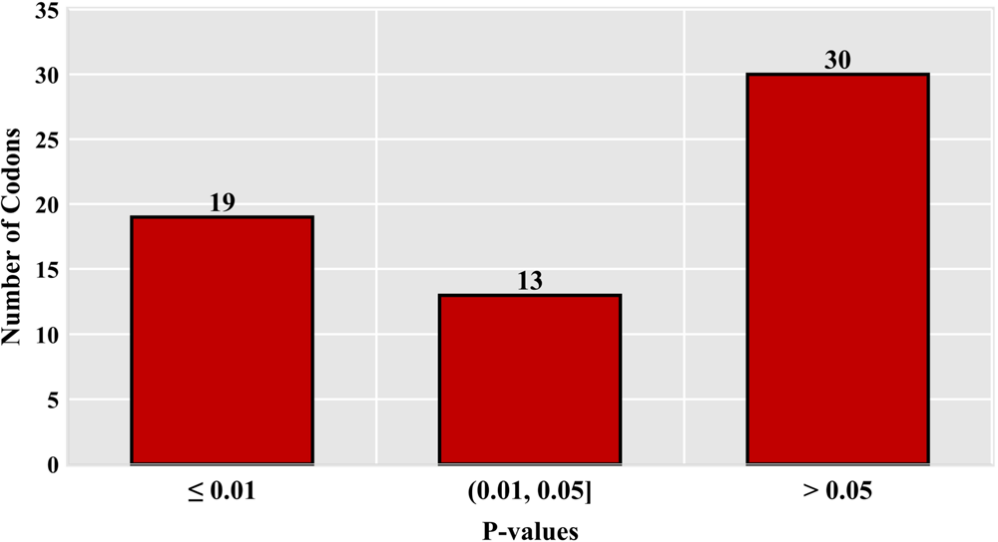
Significantly different codons. The P-values were calculated using average RSCU values of each codon in dragonflies against those in damselflies.

Moreover, to determine the exact difference in usage preference of each type of codon between dragonflies and damselflies, groups of RSCU values were compared by *t*-test (Fig. 4). As 32 codons were differently used (*t*-test with Bonferroni correction, *P* < 0.025), which is more than half (31), the usage preference patterns appeared to be formed at a broad scale, and molecular modifications might have been imposed among several codons by distinct combinations of evolutionary pressures. Seventeen of these 32 differently used codons are highly preferred, whereas 15 are less preferred codons; most are synonymous codon pairs. Changes in the direction of RSCU in each of the differently used codons were calculated as the ratio between the average RSCU in dragonflies and damselflies; except for CCA, the RSCU of highly preferred codons is higher in dragonflies than in damselflies. The codon CAA, which is the only exception, might therefore have played an important role during the molecular evolution of damselflies.

### Different mitogenomic evolutionary histories between dragonflies and damselflies as determined by the codon usage patterns

The codons are the basic units of amino acid translation. Although they code for the same amino acid, synonymous codons always have multivariate and organism-specific preference patterns, as they are not exactly identical due to multiple factors such as directional mutation and neutral selection. The distinct codon usage patterns of dragonflies and damselflies indicate that they experienced different mutations and selection pressures during molecular evolution, which are evidenced in the specific genetic adaptations of their mitochondrial genes. In the present study, we employed three approaches based on codon usage indices, namely a neutrality analysis, an ENC plot, and the ratio of synonymous to non-synonymous substitutions (Ka/Ks) to elucidate the different evolutionary mechanisms operating in the mitogenome of dragonflies and damselflies^47–51^.

**Figure 5:**
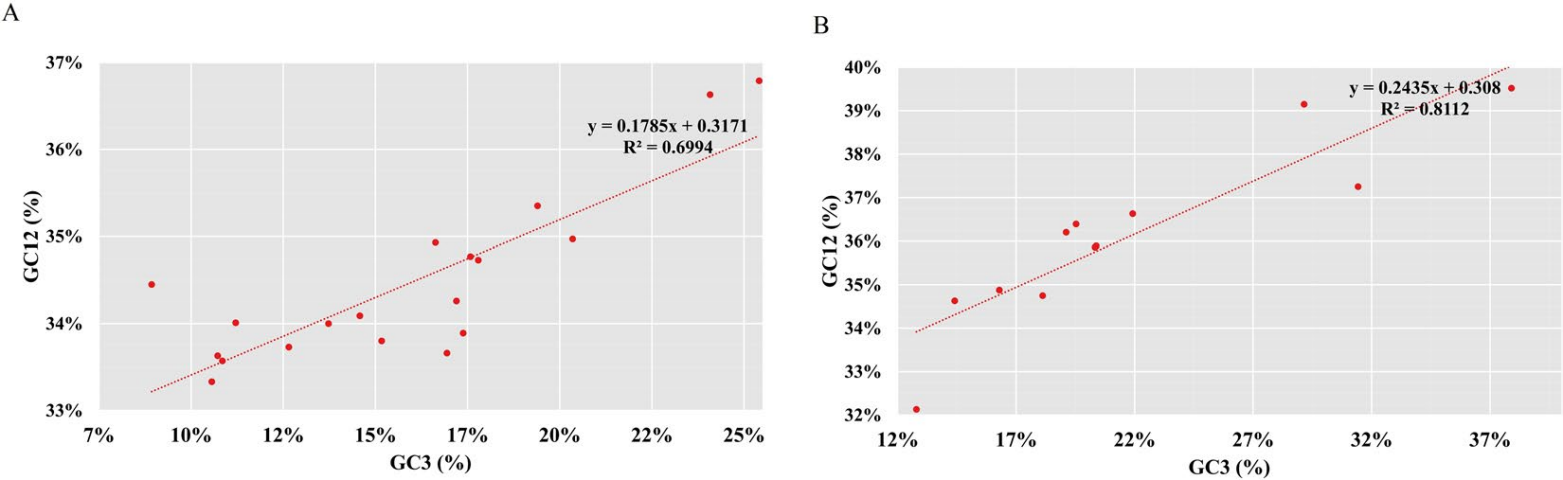
Neutrality analysis plots of dragonflies (**A**) and damselflies (**B**). A regression analysis (dotted line) was performed with the average GC content in the third codon position (GC3) against the average GC content in the first and second positions of the codons (GC12) of the mitogenome of odonates.

The neutrality analysis was performed to quantify the mutational pressure. All the codons exhibited an obvious nucleotide bias toward A and T, and the average values of GC content in the first and second positions (GC12) and in the third position (GC3) of codons were less than 40% (Fig. 5). The significant positive correlation observed between GC12 and GC3 (R^2^ = 0.6694 in dragonflies and R^2^ = 0.8112 in damselflies) suggests a directional mutation pressure acting on all codons. The slopes of the linear regressions, which were calculated to determine the exact scale of mutation pressures, were significantly less than 1 (0.1785 in dragonflies and 0.2435 in damselflies), revealing that directional mutation bias played only a minor role in the evolution of odonates. Other factors such as selection constraints from transcription and translation accounted for over 75% of the differences in codon usage bias, and therefore, shaped the evolution of mitogenomes in odonates.

**Figure 6:**
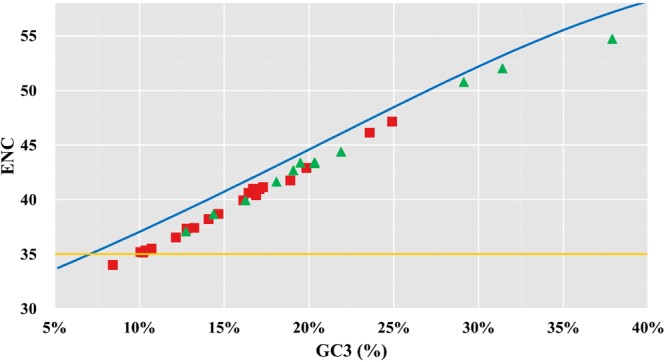
Plot analysis of effective number of codons (ENC) performed with the mitogenome of dragonflies and damselflies. The standard curve plots the relationship between the ENC and average GC content in the third codon position (GC3) in the absence of selection. Red squares represent dragonflies and green triangles represent damselflies.

The ENC plot is an effective tool to test if directional mutations are the only factor governing the codon usage pattern. The standard curve reflects the theoretical ENC values when synonymous codons are only due to directional AT or GC mutations; when codon usage is biased within AT or GC, for example when A is preferred over T, the observed ENC value will lie below the curve. All the ENC values of the species analysed in the present study were below the standard ENC curve (Fig. 6), revealing the presence of selective effects. Thus, mutations explain only part of the evolution of Odonata mitogenomes. However, the ENC values were not significantly below the standard curve, and there were no notably different deviation patterns between dragonflies and damselflies (Fig. 6). These results indicate that, although the species have various selection constraints, their usage of A- or T-ending codons are not affected. In fact, they are similarly favoured and both could be considered optimal codons that are preferentially used in dragonflies.

**Figure 7:**
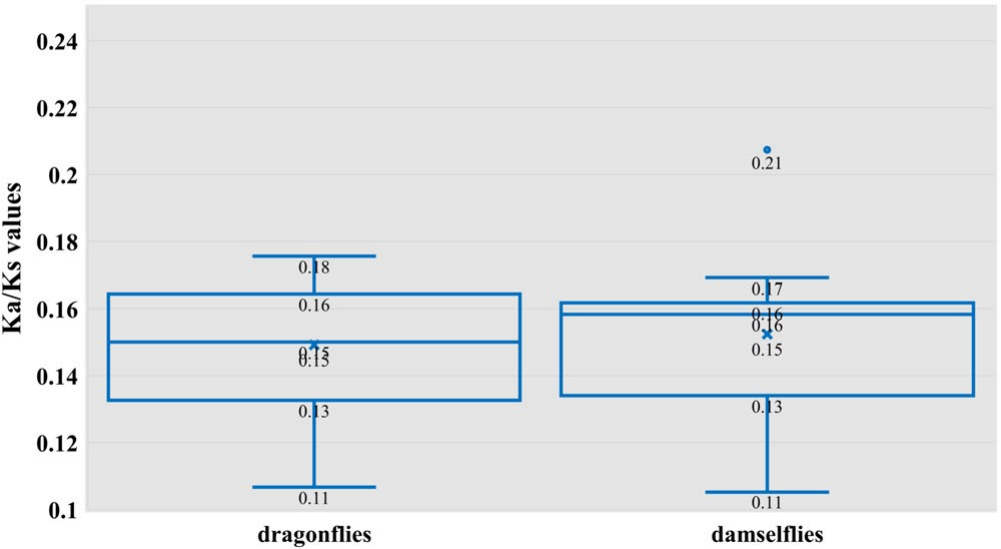
Box-plots of the ratio of synonymous to non-synonymous substitutions (Ka/Ks) in dragonflies (left) and damselflies (right). No significant difference was found (P > 0.05).

The Ka/Ks ratio is a simple measure of selection pressure on codons, which indicates neutrality (Ka/Ks = 1), negative or purifying selection (Ka/Ks < 1), and positive selection (Ka/Ks > 1). The closer the ratio to 1, the smaller the selection pressure. We calculated the Ka/Ks ratio for dragonflies and damselflies separately, and based on the Ka/Ks ratio of the ancient dragonfly *E*. *superstes* (suborder Anisozygoptera), we determined the evolutionary factors acting on their mitogenomes. The Ka/Ks ratio ranged from 0.11 to 0.18 in dragonflies and from 0.11 to 0.21 in damselflies (Fig. 7). All these values were significantly less than 1, indicating that both the groups are under intense purifying selection. As the function of purifying selection is to rapidly remove deleterious mutations, adaption effects are unlikely to affect amino acids. The protein products of mitochondrial genes are vital in the survival and thus they are more restricted in their functions. Therefore, it can be deduced that the various selection constraints occurring in the codons shape their evolution by influencing the efficiency of transcription and translation. Further comparison of the Ka/Ks ratio between dragonflies and damselflies by *t*-test confirmed this hypothesis, as we found no significant difference between the Ka/Ks ratio of the two groups (Fig. 7).

## Discussion

Although dragonflies and damselflies are attractive and fascinating groups to examine the ecology and evolution of insects, efforts to identify their genomic resources, especially mitochondrial gene sequences, have been lagging when compared with those in other insect orders^33,35,52^. To some extent, this shortage has prevented a thorough understanding of the molecular mechanisms operating in dragonflies, as well as comparative genomic analyses. Herein, we report the complete mitogenome sequence of seven dragonflies and two damselfies using an efficient next-generation sequencing method, increasing the number of complete mitogenome of damselflies available in public databases and enriching data support. Although these data will not largely solve the shortage of molecular information on Odonata and we did not identify unique genes or new arrangements, herein, we report the useful nucleotide information of two species that might be used in further analyses.

Characterising and comparing codon usage patterns between dragonflies and damselflies, which are the two main groups within Odonata, was one of the main aims of our study. The information provided herein might serve as the basis for further analyses, such as detecting different energy-consuming efforts linked to evolutionary forces in these mitogenomes. Our results revealed that the mitogenomes of Odonata have weak codon usage biases and tend to contain AT-ending codons. Such biases are a common feature in these insect mitogenomes, similar to those of fruit flies or beetles, and suggest that similar genome-changing mechanisms operated during the long-term evolution of insects. Further, codon usage patterns, as indicated by the RSCU and ENC values, seem to exhibit different trends between dragonflies and damselflies. The pattern is much more biased in dragonflies than in damselflies, and this biased pattern is specific for each type of codon. According to the premise that codon usage is an indicator of combined efforts of evolutionary forces, our results indicate that the mitogenome of these two groups are not completely neutral and might have been biased due to distinct evolutionary paths^21,42,47,48,53^. However, the exact cause of this phenomenon is still unknown. Several reports have suggested that biased usage toward preferred codons promote the efficiency of transcription and translation processes^54–56^. The codon usage bias in dragonflies suggests that they might have undergone more adaption processes and rapid changes in mitochondrial gene expression than damselflies, which increased the capacity of mitochondria metabolic processes contributing to their high flight ability. However, an alternative explanation based on different amounts of cumulative mutations is also plausible. As suggested by a previous study on the mitogenome evolution of flightless birds, strongly locomotive species rapidly remove harmful non-synonymous substitutions, indicating that they underwent strong purifying selection to maintain an efficient respiratory-chain activity^41^. During this process, synonymous substitutions were retained and directional mutations led to the usage of certain types of codons more frequently^41,50^. Based on this view, the stronger codon usage observed in dragonflies than in damselflies could have lower biological functional significance than expected, serving only as an indicator to show that they have experienced strong purifying selection. Thus, to test if the extent of codon usage reflects different metabolic processes, driving evolutionary forces have to be investigated. Further studies on transcriptional and translational mechanisms in Odonata are also required. This will clarify the relationships between codon usage and their various biological effects.

Consequently, the ENC and neutrality plots developed to detect the putative proportion of each evolutionary force revealed that both directional mutations and selection pressures contributed in shaping the mitogenome of Odonata during their evolution. Directional mutations caused strong AT-usage bias, but the dominant evolutionary force seems to be purifying selection, which rapidly eliminates deleterious adaptive changes in amino acids and restricts the effects of adaptive selection pressures at the codon usage bias level. The dominant evolutionary pressure that modified codon usage in dragonflies and damselflies appears to be purifying selection. Although a relatively stronger purifying selection could support the hypothesis that the accumulation of directional synonymous substitutions drives codon usage, this was not observed in dragonflies. Thus, the codon usage patterns seem to be linked to gene expression mechanisms in the mitogenome of Odonata and somehow governed their molecular evolution. Our results agree with the generally observed weaker flight performance and predatory abilities of damselflies than those of dragonflies. Although the codon usage pattern in consistent with the difference is flight ability, to confirm the relationship, a method needs to be identified that actually quantifies species flight ability and collect these data from more species (now it is just a general observation that dragonflies are better fliers than damselflies). The expression data of mitochondrial genes between codon usage and expression level in Odonata are also required to establish the connection. Both dragonflies and damselflies are big clades; the 31 species included in the present study represent sparse sampling of species diversity, and mitochondrial genome sequences of more species are needed. Without the direct support of expression data and without examining more taxa, we can only suggest that functional adaptations conditionally occurred at some sites and that codon usage might have affected expression efficiency in Odonata mitogenomes. A more efficient and stabilised protein production process in the mitogenome of dragonflies is therefore suggested. This can explain their remarkably developed locomotion ability compared with that of damselflies.

## Methods

### Sample collection and genomic DNA extraction

Wild adult specimens of *P*. *foliacea* and *A*. *atratum* were collected in Chang’an County, Xi’an, China. The samples for *Tramea virginia*, *Orthetrum testaceus*, *Orthetrum sabino*, *Orthetrum melania*, *Deielia phaon*, *Acisoma panorpoides*, *Trigomphus carus* were collected from Taizhou city, Zhe-Jiang province, China. Five individuals of each species were collected. All specimens were identified to species level, preserved in 100% ethanol, and stored at −80 °C. The genomic DNA was extracted from the whole body of each individual sample using the Tiangen DNA kit (Tiangen Biotechnology, Beijing, China) and used for Illumina HiSeq (Illumina, San Diego, CA, USA) sequencing at Genesky (Shanghai, China).

### Sequencing, assembly, and annotation of mitogenomes

Approximately 500 ng of the total genomic DNA was used to prepare 250-bp pair-end libraries using the NEBNext Modules (New England Biolabs, Ipswich, MA, USA). Sequencing of complete mitogenomes was performed from both ends using the whole-genome Illumina HiSeq2500 platform. Overall, the raw reads of 300 bp were obtained for all nine odonates, which were quality-trimmed with Trimmomatic 0.35^57^ and used to assemble mitogenomes in the Assembly by Reduced Complexity (ARC) pipeline (http://ibest.github.io/ARC/)^58^. This pipeline implements a hybrid mapping and assembly approach for targeted assembly of homologous sequences. Two mitogenomic fragments (GenBank: KX161841 for dragonflies and KC878732 for damselflies) were used as the initial reference. The individual mitochondrial reads were generated for these odonates. The mitogenomes were annotated using the MITOS Web Server (http://mitos2.bioinf.uni-leipzig.de/index.py)^59^, and adjusted using other available Odonata mitogenomes. The annotated mitogenomic sequences have been deposited in GenBank (https://www.ncbi.nlm.nih.gov/genbank) and assigned the accession numbers of MH751435-MH751441 (dragonflies) and KP233804-KP233805 (damselflies).

### Data analysis

The complete mitogenome sequence of 22 odonates was obtained from GenBank (Supplementary Table S2). Along with our data, 13 complete PCGs were extracted from the 31 sequences and concatenated into a single dataset using MEGA7^60^. This dataset was further aligned using MAFFT5^61^ in the codon alignment mode and the stop codons were removed. The final dataset comprised 88,900 codons, with codon number ranging from 3604 to 3741.

The indices of codon usage and synonymous codon usage bias^62^ were measured using CodonW 1.4.2^48^. These included GC (GC12 and GC3), ENC, and RSCU. The ENC was used to measure the degree of codon bias, which negatively correlated with codon usage bias, with values varying from 20 to 61. When ENC = 20, there was an absolute bias toward a synonymous codon, whereas ENC = 61 indicates neutral codon usage (i.e., even usage of all codons)^49^. The RSCU is the ratio between the observed frequency of a synonymous codon and the expected frequency of that codon when all codons are used evenly for that particular amino acid. Thus, the RSCU directly exhibits the deviation of synonymous codon usage from their even usage. RSCU > 1 indicates codon-usage preference, RSCU < 1 indicates a less-frequent usage of that codon, and RSCU = 1 indicates no bias in codon usage^47^.

The *t*-test was used to analyse differences in the ENC and RSCU using SPSS 22.0 (IBM, New York, NY, USA), considering *P* < 0.025 as the significance threshold. The data were sufficiently normally distributed to allow *t*-test analysis. A hierarchical cluster analysis was performed with the RSCU values by Spearman rank method using Cluster 3.0^63^. A correspondence analysis between GC12 and GC3, which is known as the neutrality plot, was performed using the SPSS 22.0 to estimate directional mutation bias. An ENC plot, representing the deviation of observed ENC values from the standard expected ENC curve, was obtained using DAMBE 5.0^64^. The ratio of non-synonymous to synonymous substitutions (Ka/Ks) was calculated using PamlX 1.3.1^65^, with a mitochondrial DNA sequence of the ancient dragonfly *E*. *superstes* as reference.

## Electronic supplementary material

Dataset 1
